# Data on aluminum concentration in drinking water distribution network of rural water supply in Sistan and Baluchistan province, Iran

**DOI:** 10.1016/j.dib.2018.08.180

**Published:** 2018-09-05

**Authors:** Hesam Akbari, Hamed Soleimani, Majid Radfard, Hamed Biglari, Hossein Faraji, Samira Nabavi, Hamed Akbari, Amir Adibzadeh

**Affiliations:** a Health Research Center, Life Style Institute, Baqiyatallah University of Medical Sciences, Tehran, Iran; b Department of Environmental Health, School of Public Health, Tehran University of Medical Sciences, Tehran, Iran; c Department of Environmental Health Engineering, School of Public Health, Gonabad University of Medical Sciences, Gonabad, Iran; d Students Research Committee, Hamadan University of Medical Sciences, Hamadan, Iran

**Keywords:** Aluminum, Groundwater resources, Distribution network of drinking water, Geographic information system (GIS), Sistan and Baluchistan

## Abstract

The aim of this study is to determine the Aluminum concentration in groundwater resources of Sistan and Baluchestan province, Iran. See the data in this article. For the purpose of this study, a total of 871 water samples were collected and values of quality parameters including pH, turbidity, total dissolved solids (TDS) and Aluminum concentration were measured (with two-time repetitions) according to Standard Methods for the Examination of Water and Wastewater, during the year 2016. The mean, maximum, minimum of Aluminum concentrations in all groundwater resources of Sistan and Baluchistan province, were: 0.015, 0.059, 0.0004 mg/l, respectively and also, the standard deviation was 0.012. Moreover, employing GIS software, the geo-statistical distribution of Aluminum concentration in groundwater aquifer in Sistan and Baluchestan was mapped.

**Specifications table**

**Table:** 

Subject area	Environmental Sciences
More specific subject area	Heavy metal (Aluminum)
Type of data	Tables, Figures
How data was acquired	The pH and temperature parameters were measured by pH meter and turbidity meter, respectively. Also the measurement of the Aluminum concentration levels in the water samples was carried out using Atomic Absorption device (Analytic Jena AA6 vario 6).
Data format	Raw, Analyzed
Experimental factors	Determine the concentration levels of Aluminum
Experimental features	Water samples were carried out using Atomic Absorption device (Analytic Jena AA6 vario 6).
Data source location	Sistan and Baluchistan, Iran
Data accessibility	The data are available with this article
Related research article	M.Radfard, M.Yunesian, R. Nabizadeh Nodehi,H. Biglari, M. Hadi, N.Yosefi,M.Yousefi,A. Abbasnia, AH. Mahvi. Drinking water quality and Arsenic health risk assessment in Sistan-and-Baluchestan, Southeastern province Iran. Human and Ecological Risk Assessment: An International Journal (2018) (DOI:10.1080/10807039.2018.1458210).

**Value of the data**

• Determination of the water parameters including Al, pH, TDS, Turbidity in ground water resources was conducted in Sistan and Baluchistan province, Iran.
• Data with Arc Gis zoning can help to better understanding the quality of ground water in this area.
• According to national standards, the concentration levels of Aluminum were within the standard range during the studied period. Therefore, consumption of water resources of this area likely will not cause any health problems associated with Aluminum metal.

## 1 Data

**Fig. 1:**
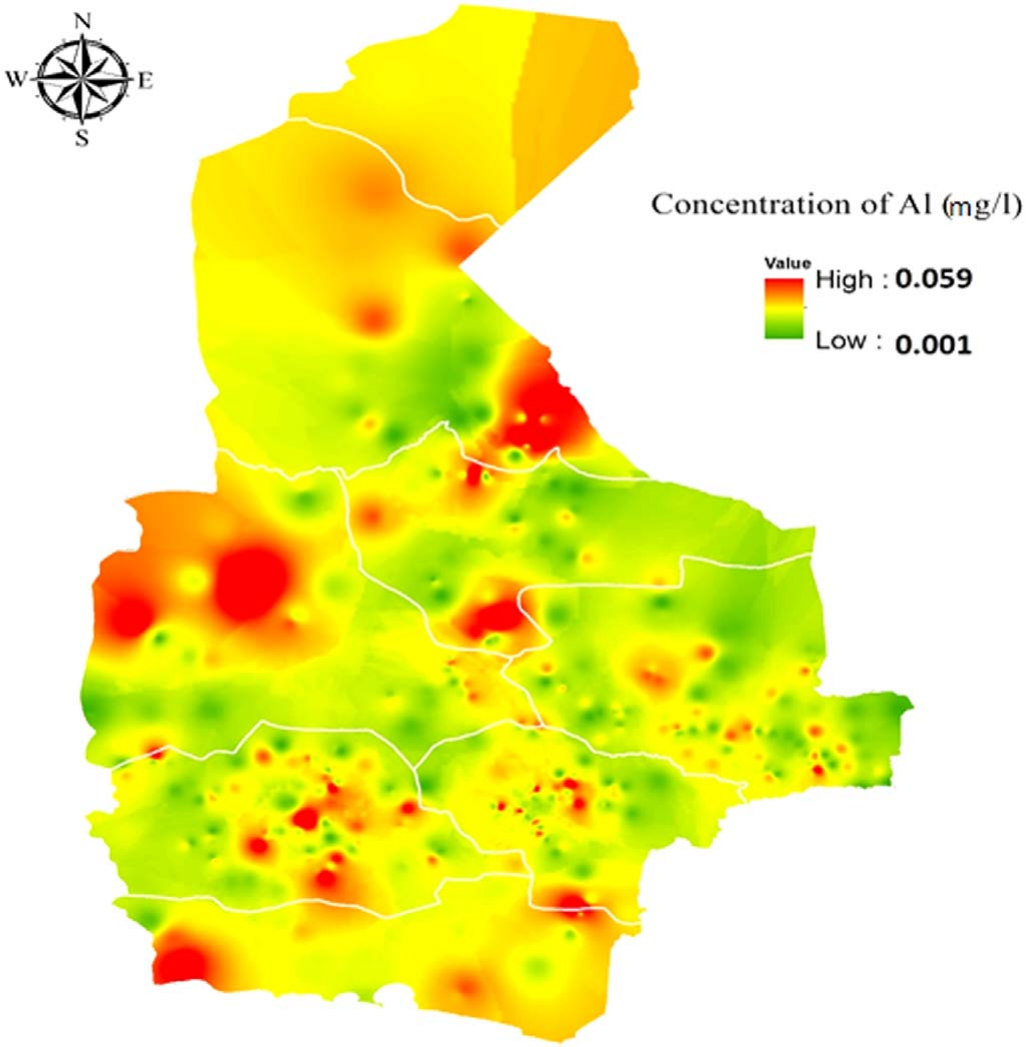
Dispersion of Al concentration (mg L^−1^) by GIS software.

Tables 1 and 2 shows the minimum, mean, maximum and standard deviation of studied parameters including Al, pH, TDS, and Turbidity in the groundwater of the different cities (8 cities) of the Sistan and Baluchistan province. Also Fig. 1 illustrates the geo-statistical.

**Table 1:** Minimum, mean, maximum and standard deviation of Al and pH in different cities of the province.

City	Number	Al (mg L^−1^)				pH			
		Min	Average	Max	STDEV	Min	Average	Max	STDEV
Iranshahr	124	0.001	0.016	0.059	0.018	6.85	7.88	8.58	0.346
Chabahar	23	0.004	0.012	0.021	0.005	7.37	7.805	8.27	0.236
Khash	69	0.001	0.014	0.041	0.009	7.04	7.373	8.35	0.267
Zahedan	54	0.001	0.016	0.042	0.013	4.21	7.894	8.3	0.247
Zabol	16	0.001	0.015	0.036	0.014	7.14	7.892	8.15	0.234
Saravan	247	0.001	0.014	0.059	0.013	6.81	7.816	8.3	0.285
Sarbaz	23	0.001	0.013	0.042	0.01	7.34	7.772	8.25	0.197
Konarak	113	0.001	0.013	0.038	0.011	7.22	7.706	8.2	0.3
Nikshahr	202	0.001	0.019	0.048	0.014	7.09	8	8.37	0.227

**Table 2:** Minimum, mean, maximum and standard deviation of TDS and Turbidity in different cities of the province.

City	Number	TDS(mg L^−1^)				Turbidity(NTU)			
		Min	Mean	Max	STDEV	Min	Average	Max	STDEV
Iranshahr	124	241	1049.47	3130	570.82	0.19	0.638	4.28	0.589
Chabahar	23	452	1313.61	2426	682.61	0.32	0.638	3.02	0.559
Khash	69	439	1091.04	2573	524.09	0.19	0.684	4.67	0.878
Zahedan	54	306	1870.28	9001	1178.74	0.2	0.699	5.7	0.991
Zabol	16	425.6	546.4	702.72	388.19	0.23	0.917	1.6	0.451
Saravan	247	114	934.5	2413	453.87	0.18	0.904	5.45	0.844
Sarbaz	23	274	548.35	1965	226.43	0.16	0.913	7.5	1.148
Konarak	113	956	1314.78	2035	329.61	0.23	0.842	4.46	0.957
Nikshahr	202	312	708.46	1542	273.84	0.2	0.637	3.65	0.557

Distribution of Al concentration. In addition to, the mean of the total parameters in the studied area presented in Table 3.

**Table 3:** Mean of total parameters in the province.

Parameter	Al(mg L^−1^)	pH	Turbidity(NTU)	TDS(mg L^−1^)
Max	0.059	8.31	4.5	2485.3
Min	0.0004	7.11	0.211	391.07
Average	0.015	7.8	0.763	1033.88
STDEV	0.012	0.271	0.774	514.02
More than the permissible	0%	0%	11%	79%
Number	871	871	871	871

## 2 Experimental design, materials and methods

### 2.1 Study area description

**Fig. 2:**
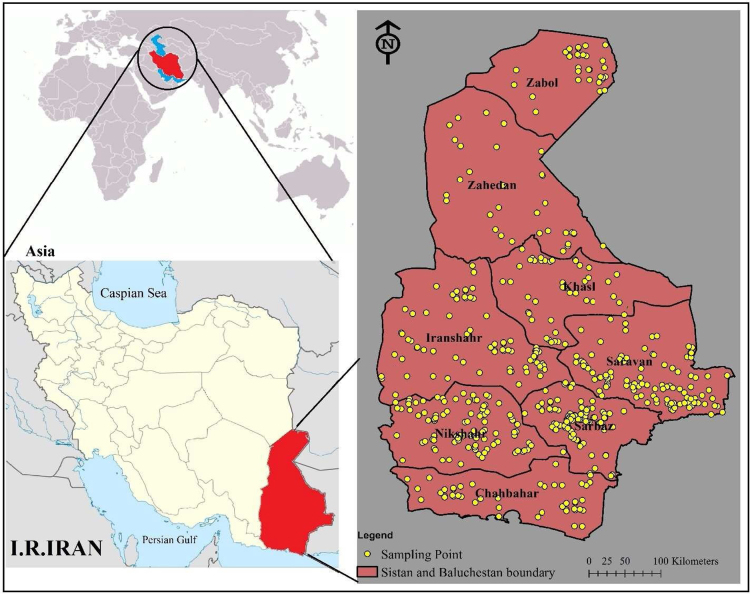
Location map of the studied area and sampling site.

Sistan and Baluchistan province one of the large province of Iran, located province between 58°55׳- 63°20’ eastern longitude and 25°4׳- 31°25׳ northern latitude, south of Iran [1] (Fig. 2).

### 2.2 Sample collection and analytical procedures

This study was conducted in Iranshahr, Chabahar, Khash, Zahedan, Zabol, Saravan, Sarbaz, Konarak, Zehak and Nik Shahr counties in Sistan and Baluchestan province. In the year 2017. Sampling was done from the water wells directly and Also, In the lack of the direct access to the wells, samples were taken from the closest water distribution network that fed from wells. The GIS software and IDW interpolation method were used to determine the latitude and longitude of well locations [6], [7], [8], [9], [10], [11], [12]. The location of the counties, the sampling sites and the dispersion of the Aluminum is shown in the Fig. 1. After surveying the studied area, 871 major water resources that were used for bathing, washing, and mainly for drinking, were selected within the 1-year-monitoring period and then mapped using the GIS software [10], [11], [12]. Samples were collected using a plastic sampling containers, which were washed with 20% Nitric acid solution. Also, in order to protect samples containers from secondary pollution, they were preserved with plastic bags on the transportation to sampling sites. Finally, to prevent microbial activity, the samples were stored in a Polystyrene box at 4° C and also, 2 ml Nitric acid was added to each one liter of samples to increase the stability of the them [12]. The pH and Turbidity parameters were measured at sampling site and measurement of TDS and Aluminum samples were performed according to Standard Methods for the Examination of Water and Wastewater in the laboratory [13]. To ensure the accuracy of the data, experiments were repeated after one week and also, it should be noted that sampling for repetition of the experiments, was don from the same sampling site. Finally, the data was analyzed using independent t-test and also, Excel 2007 software. GIS was used to plot the geostatistical distribution of Aluminum, and additionally, to identify the areas with maximum level of pollutants [2], [3], [4], [5]. The pH and temperature were measured by pH meter and turbidity meter, respectively. Also, the measurement of the Aluminum concentration levels in the water samples was carried out using Atomic Absorption device (Analytic Jena AA6 vario 6) [14].
